# Is fasting safe? A chart review of adverse events during medically supervised, water-only fasting

**DOI:** 10.1186/s12906-018-2136-6

**Published:** 2018-02-20

**Authors:** John S. Finnell, Bradley C. Saul, Alan C. Goldhamer, Toshia R. Myers

**Affiliations:** 1 AOMA Graduate School of Integrative Medicine, Austin, TX 78745 USA; 2 NoviSci, Durham, NC USA; 3 TrueNorth Health Center, Santa Rosa, CA 95404 USA; 4 TrueNorth Health Foundation, 1501 Pacific Avenue, Santa Rosa, CA 95404 USA

**Keywords:** Fasting, Safety, Adverse events

## Abstract

**Background:**

Evidence suggests that fasting, during which only water is consumed, results in potentially health promoting physiological effects. However, peer-reviewed research assessing the safety of water-only fasting is lacking. To address this, we conducted a chart review to describe adverse events (AEs) that occurred during medically supervised, water-only fasting.

**Methods:**

Electronic charts from patient visits to a residential medical facility from 2006 to 2011 were reviewed. Patients who were at least 21 years of age and water-only fasted for ≥2 consecutive days with a refeeding period equal to half of the fast length were included. Out of 2539 charts, 768 visits met our inclusion and exclusion criteria. AEs were abstracted from chart notes and classified according to CTCAE (v4.03) and MedDRA (v12.1) terminology. Descriptive analysis of AEs is reported.

**Results:**

During the protocol period, the highest grade AE (HGAE) in 555 visits was a grade 2 event or lower, in 212 visits it was a grade 3 event, in 1 visit it was a grade 4 event, and there were no grade 5 events. There were 2 (0.002%) visits with a serious adverse event (SAE). The majority of AEs identified were mild (*n* = 4490, 75%) in nature and known reactions to fasting.

**Conclusions:**

To our knowledge, this is the most comprehensive analysis of AEs experienced during medically supervised, water-only fasting conducted to date. Overall, our data indicate that the majority of AEs experienced were mild to moderate and known reactions to fasting. This suggests that the protocol used in this study can be safely implemented in a medical setting with minimal risk of a SAE.

**Electronic supplementary material:**

The online version of this article (10.1186/s12906-018-2136-6) contains supplementary material, which is available to authorized users.

## Background

Modern humans evolved to survive periods of food shortage and have voluntarily fasted for at least 2000 years. Fasting is broadly defined as the voluntary abstinence of some or all caloric foods and beverages for therapeutic, spiritual, or political reasons [1]. In the last century, research in animals and humans has uncovered several potentially health-promoting physiologic responses to fasting including ketogenesis, hormone modulation, reduced oxidative stress and inflammation, and increased stress resistance, lipolysis, and autophagy [1–4]. Clinical research in humans also indicates that fasting improves hypertension, [5, 6] rheumatoid arthritis, [7] cardiovascular disease, [8, 9] metabolic syndrome, [10, 11] osteoarthritis, [12] fibromyalgia, [13] chronic pain, [14] and quality of life [15].

Wilhelmi de Toledo et al. recently reviewed several fasting methods and established the first peer-reviewed therapeutic fasting guidelines in English (revision of the 2002 German publication [16, 17]). The guidelines specify indications, contraindications, and methods for safe implementation and are based on the Buchinger Method (a modified diet supplying 250–500 kcal/day in the form of vegetable broth, fruit and vegetable juices, and honey). The guidelines are also largely applicable to water-only fasting, which is defined as the complete abstinence of substances except for pure water [18] and is an established method of therapeutic fasting [1–3, 19, 20]. Yet it is only briefly described by Wilhelmi de Toledo et al. as a “zero calorie diet” that has not been practiced since the 1970s, when it was primarily used for weight reduction [17].

Therapeutic water-only fasting research did decline following a period in the 1960s and early 1970s when the method was used as an obesity treatment, [21–24] in part due to reports of serious complications, including death, in some fasting subjects [25–30]. During this time, it was common to arbitrarily implement fasts lasting 60 days or longer [29, 31, 32] and to administer chemical compounds to facilitate prolonged fasts. [25, 28, 33, 34]. Additionally, physicians and researchers did not always screen subjects for contraindications, terminate fasts upon complication, or properly refeed subjects, [25, 26, 28, 29] all of which are necessary for the safe implementation of water-only fasts [1–3]. Therefore, it is difficult to conclude if water-only fasting is inherently dangerous or if the complications were caused by unintentionally harmful fasting practices.

Despite the documented decline in fasting research and contrary to claims by Wilhemi de Toledo et al., [17] water-only fasting was not entirely discontinued in the 1970s. It has since been utilized for many therapeutic purposes such as the treatment of hypertension, [5, 6] cardiovascular disease, [9] appendicitis, [35] follicular lymphoma, [36] and as an adjunct to chemotherapy [37]. Importantly, previous therapeutic implementation and clinical research led to the development of a water-only fasting protocol (see Methods) that safeguards against serious complications and minimizes minor reactions [1–3]. Nevertheless, there is a lack of objective, peer-reviewed data on the safety of water-only fasting.

Therefore, we were motivated to systematically assess the safety of medically supervised, water-only fasting. To this end, we reviewed and characterized the adverse events (AEs) experienced by patients who water-only fasted at TrueNorth Health Center (TNHC) over a five-year period using Common Terminology Criteria for Adverse Events (CTCAE v4.03 [38]) and Medical Dictionary for Regulatory Activities (MedDRA v12.1 [39]). We report the severity, frequency, and nature of patient AEs that occurred during this time.

## Methods

### Setting

TNHC (www.truenorthhealth.com) is an integrative medical facility in Santa Rosa, CA, USA that offers a residential health education program specializing in water-only fasting and dietary intervention. Patients in this study had access to medical, naturopathic, and chiropractic doctors. Private rooms, 24-h medical supervision, and daily educational activities were also provided.

### Water-only fasting protocol

The detailed water-only fasting protocol used in this study is within the standards established by the International Association of Hygienic Physicians (Additional file 1: Supplementary Methods) [40]. Briefly, patients electing to water-only fast at TNHC routinely underwent a comprehensive physical, neurological, and psychological examination including medical history, urinalysis, complete blood count with differentials, and comprehensive metabolic panel. Patients with contraindications or taking medications that could not be discontinued, with the exception of thyroid medications which can be continued at a reduced concentration, were not admitted for water-only fasting.

Patients were instructed to eat a diet of fresh raw fruits and vegetables and steamed starchy vegetables for at least 2 days before fasting. During the fast, patients remained on site, drank a minimum of 40 ounces of distilled water per day, and minimized physical activity. Medical staff monitored fasting patients twice daily and recorded vital signs and symptoms into chart notes. Urinalysis and the aforementioned blood tests were repeated weekly or as directed by a clinician. Water-only fasts were discontinued when symptoms stabilized, the patient requested termination of the fast, or the clinician deemed it necessary for medical reasons.

Following the fast, patients refed for a period of time lasting half of the fast length. Standard refeeding consisted of five phases (1 phase for each 7–10 days of water-only fasting) (Supplementary Methods), beginning with juice and followed by gradual introduction of solid plant foods (free from added sugar, oil, and salt) (Additional file 1: Supplementary Methods). Once patients were eating solid foods, the gradual reintroduction of moderate exercise was allowed. During the refeeding process, patients were offered twice-daily monitoring by clinicians.

### Database design and study population

Electronic charts from May, 2006 to December 31, 2011 were extracted into a single relational database (MS Access). The data collected included age, gender, chief complaints, treatment status (i.e., water, juice, broth, or food), and clinical chart notes. Chief complaints data were derived from self-reports and clinical diagnoses.

**Fig. 1:**
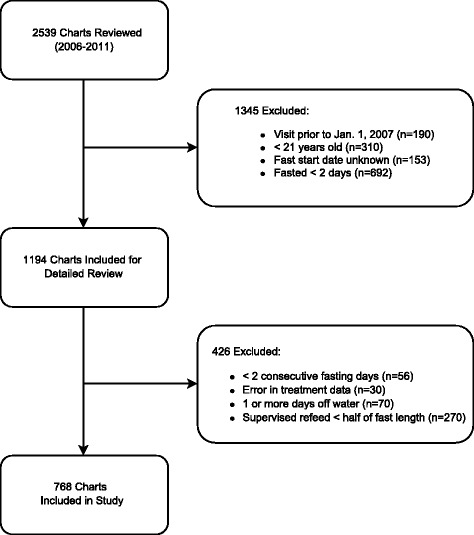
Study population flow chart

The study included visits by patients who water-only fasted for at least two consecutive days and refed for at least half of the fast length. The study excluded visits by patients who fasted prior to Jan. 1, 2007 because of incomplete data records, were under twenty-one years old, water-only fasted for one day or less because this length is defined as an intermittent fast and generally does not require medical supervision, [41] interrupted the water-only fast with any amount of vegetable broth or juice and/or fruit juice, had supervised refeeding less than half of the fast length, or if there were insufficient data to determine treatment status on a given day (Fig. 1). Out of 2539 visits, 768 were included in our study. Of the selected visits, the analysis dataset included visit days from the day fasting began to day the refeeding period (1/2 length of fast post-fast) ended. For fasts of an odd number of days, the refeeding period was rounded up to the nearest integer (e.g., if a visit had 3 days of fasting, then analysis data included 2 days of refeeding). There was no information about the condition of the patient other than treatment status for 5.2% (496/9570) of total visit days (312 during fasting/184 refeeding).

### Adverse event identification and coding

Data on AEs were collected from clinical chart notes of self-reported symptoms, clinical and diagnostic findings, and medical management of symptoms. Laboratory values were not systematically included due to data incompleteness. The CTCAE (v4.03) scale was used to grade AE severity: AE1 – mild, AE2 – moderate, AE3 – severe, AE4 – life threatening or disabling, and AE5 – death. AEs were characterized using the MedDRA (v12.1) terminology according to the system organ class (SOC), which uses the highest level term (HLT), including anatomical or physiological system, etiology, and/or purpose, and the lowest level term (LLT). Pain-related AEs, reported on a visual-analog scale (VAS) from 0 to 10, were reported as follows: AE1 = VAS 1–4, AE2 = VAS 5–7, and AE3 = VAS 8–10 [42, 43]. A serious adverse event (SAE) was determined based on the definition used by the Department of Health and Human Services [44]. A codification guide was created to ensure that there was consistent codification of AEs. A single trained clinician (J.S.F) identified, reviewed, and classified AEs, according to the above criteria, for each daily entry in the electronic chart note. A user interface within the relational database (MS Access) was used for direct data entry of the codified data. One week after the conclusion of data abstraction, intra-rater reliability was assessed based on a random sample of daily logs. The dataset is available from the Dryad repository, 10.5061/dryad.6cg6j.

### Intra-rater consistency

Inter-rater reliability was assessed using a blind, random sample of daily logs (*n* = 645, 7%). There was 80.5% agreement between MedDRA codes identified in the original coding and secondary assessment. Of these, the weighted kappa statistic (squared weights) measuring agreement of the AE grade was 0.92 [45].

### Adverse event descriptive analysis

Visits were stratified by water-only fast length: 2–7 (short), 8–14 (medium), 15–21 (long), or 22+ (extended) days. These categories were chosen to represent lengths of typical fasts. Within visits, days were grouped by fasting and refeeding status. Summary statistics of the study population at the patient and visit levels were tabulated. Chief complaints were categorized by SOC when possible. For each visit, the highest grade AE (HGAE) experienced (of any MedDRA term) and the total AEs were calculated for the fasting, refeeding, and entire protocol periods. Tallies and percentages of HGAE within each of these periods were computed in total and by the fasting length strata. Spearman’s correlation between fasting length strata and HGAE was computed using integer scores for the fasting length strata. For each MedDRA term, counts and percentages of visits with at least one AE of a term were computed for each AE grade and for all AE grades combined. Similar counts and percentages were computed for individual AEs without taking visits into account. We report MedDRA terms that were experienced in more than 10%. Association between a visit’s HGAE and age was assessed graphically and measured with Spearman’s correlation [46]. An association between HGAE and gender was tested using a two-sided Fisher’s exact test. The paired difference in HGAE during fasting versus refeeding was tested using a paired t-test. Kaplan-Meier estimates of cumulative incidence (CI) were computed for each category of fasting length for the following AE categories: any AE, any AE ≥ 2, and any AE ≥ 3 [47]. All AEs of grade 3 or higher were carefully reviewed (A.C.G., T.R.M, J.S.F.), and SAEs were further described by brief narrative. Analyses were performed in R 3.1 using the survival, [48, 49] irr, [45] ggplot2, [50] dplyr, [51] and htmlTable packages [52].

## Results

### Patient demographics and visit characteristics

We analyzed charts from 768 visits of patients who water-only fasted at TNHC from 2007 to 2011 for at least 2 consecutive days followed by a refeeding period equal to half of the fast length. There were a total of 652 patients, 409 (63%) were female, and the median (interquartile range, IQR) age at first visit was 55 (17.25) years old. Of these, 54 patients fasted 2 times and 23 fasted 3 or more times during the study period.

There were 768 visits, comprising 9570 visit days and 6265 fasting days. The median fasting length was 7 (IQR 6) days; the shortest fast was 2 days and longest fast was 41 days. Of these, there were 446 short (2–7 fasting days, median [IQR] = 5 [3] days), 238 medium (8–14 fasting days, median [IQR] = 10 [3] days), 64 long (14–21 fasting days, median [IQR] = 18 [4] days), and 20 extended (22 or more fasting days, median [IQR] = 27.5 [6.2] days) visits. Table 1 describes chief complaint categories as total counts and as a percentage of total visits. A patient could have had more than one chief complaint per visit. Quality of life, including prevention and fatigue, was the primary reason patients visited TNHC (*n* = 384, 50%). Other major chief complaint categories included the cardiovascular (*n* = 193, 25.1%), musculoskeletal (*n* = 147, 19.1%), gastrointestinal (*n* = 122, 15.9%), and endocrine (*n* = 107, 13.9%) systems. Following prevention (*n* = 358, 46.7%), hypertension (*n* = 152, 19.7%) was the largest chief complaint category.

**Table 1:** Chief complaints

	*n* (% of visits)^a^		*n* (% of visits)
Quality of life Prevention (358), Fatigue (40), Fasting (2)	384 (50)	Autoimmune Rheumatic (22), Lupus (16), Sjogren’s (3)	43 (5.6)
Cardiovascular Hypertension (152), Coronary Artery Disease (11), Arrhythmia (8)	193 (25.1)	Dermatological Rash (9), Psoriasis (8), Eczema (8)	42 (5.5)
Musculoskeletal Arthritis (45), Osseous (36), Myalgia (21)	147 (19.1)	Cancer Breast (11), Andrologic (10), Lymphoma (3)	36 (4.7)
Gastrointestinal Colitis (23), Gastritis (20), Constipation (17)	122 (15.9)	Substance abuse Drug (17), Food (12), Meds (4)	32 (4.2)
Endocrine Thyroid (52), Diabetes (46), Reproductive (10)	107 (13.9)	Dietary Nutrition (9), Allergies (9), Eating disorder (7)	25 (3.3)
Metabolic Dyslipidemia (59), Dysglycemia (11)	66 (8.6)	Infection Fungal (6), Bacterial (6), Viral (5)	24 (3.1)
Neurological Headache (31), Neuropathy (16), Dizziness (6)	62 (8.1)	Unknown Other (20)	20 (2.6)
Genitourinary Gynecologic (25), Urologic (18), Renal (8)	57 (7.4)	Tumor Neck (6), Breast (1), Colon (1)	8 (1)
Psychiatric Depressive (21), Anxiety (12), Attention (9)	57 (7.4)	Immune Inflammation (6)	6 (0.8)
Pulmonological Inflammation (27), Dyspnea (19), Infection (8)	56 (7.3)	Laboratory value Abnormal (5)	5 (0.7)
Ear, Eyes, Nose, & Throat Sinus (18), Allergies (15), Ocular (13)	52 (6.8)	Environmental Exposure (3)	3 (0.4)

^a^Patients may have indicated more than one chief complaint per visit

### Adverse events experienced during fasting and refeeding

AEs were classified on a graded scale of 1–5 according to CTCAE (v4.03) and MedDRA (v12.1) criteria (see Methods). HGAE experienced during the fasting, refeeding, and entire protocol period of each visit is presented in Table 2. During the entire protocol period (i.e., fasting and refeeding) for all visit lengths, the HGAE experienced in the majority of visits (*n* = 555, 72.3%) was grade 2 or lower and in 212 visits (26.6%) it was grade 3. There was a weak positive correlation between fast and refeed length and HGAE (Table 2). There was 1 patient with a grade 4 AE during a medium visit. There were no deaths (AE5) during any visits. There was a weak positive correlation between HGAE and fast duration (Spearman’s ρ = 0.28, *p* = < 0.001) and a weak positive correlation between HGAE and age (Spearman’s ρ = 0.11, *p* = 0.002) (Additional file 1: Figure S1a, b). There was no correlation between HGAE and gender (*p* = 0.628) (Additional file 1: Figure S1c). During refeeding, the mean HGAE was 0.56 less than during fasting (paired t = − 16.28, *p* = < 0.001).

**Table 2:** Highest Grade AE experienced by duration of fast

AE Grade, *n* (%)						
	none	1	2	3	4	5
Fasting (Spearman’s ρ = 0.24, *p* < 0.001)						
Short	62 (13.9)	136 (30.5)	159 (35.7)	89 (20)	0 (0.0)	0 (0.0)
Medium	10 (4.2)	61 (25.6)	108 (45.4)	58 (24.4)	1 (0.4)	0 (0.0)
Long	0 (0.0)	7 (10.9)	32 (50)	25 (39.1)	0 (0.0)	0 (0.0)
Extended	0 (0.0)	2 (10)	6 (30)	12 (60)	0 (0.0)	0 (0.0)
Total	72 (9.4)	206 (26.8)	305 (39.7)	184 (24)	1 (0.1)	0 (0.0)
Refeeding (Spearman’s ρ = 0.23, *p* < 0.001)						
Short	133 (29.8)	181 (40.6)	98 (22)	34 (7.6)	0 (0.0)	0 (0.0)
Medium	39 (16.4)	108 (45.4)	69 (29)	21 (8.8)	1 (0.4)	0 (0.0)
Long	2 (3.1)	24 (37.5)	25 (39.1)	13 (20.3)	0 (0.0)	0 (0.0)
Extended	0 (0.0)	7 (35)	8 (40)	5 (25)	0 (0.0)	0 (0.0)
Total	174 (22.7)	320 (41.7)	200 (26)	73 (9.5)	1 (0.1)	0 (0.0)
Entire period (Spearman’s ρ = 0.22, *p* < 0.001)						
Short	43 (9.6)	125 (28)	175 (39.2)	103 (23.1)	0 (0.0)	0 (0.0)
Medium	7 (2.9)	58 (24.4)	106 (44.5)	66 (27.7)	1 (0.4)	0 (0.0)
Long	0 (0.0)	3 (4.7)	32 (50)	29 (45.3)	0 (0.0)	0 (0.0)
Extended	0 (0.0)	1 (5)	5 (25)	14 (70)	0 (0.0)	0 (0.0)
Total	50 (6.5)	187 (24.3)	318 (41.4)	212 (27.6)	1 (0.1)	0 (0.0)

Short, 2–7 fasting days; Medium, 8–14 fasting days; Long, 15–21 fasting days; Extended, 22+ fasting days

**Fig. 2:**
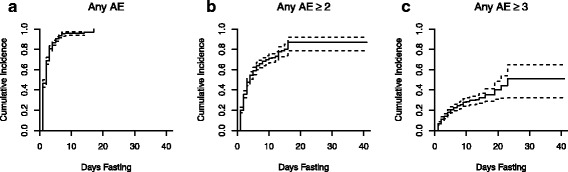
Cumulative incidence of adverse events by fast length. Kaplan-Meier curves (solid line) and 95% confidence intervals (dashed line) for (**a**) any AE; (**b**) any AE greater than or equal to 2 and; (**c**) any AE greater than or equal to 3

Kaplan-Meier CI plots for AEs during fasting and during refeeding are shown in Fig. 2. At day 5 of fasting, the estimated cumulative incidence (ECI) for experiencing any AE was 0.90 (95% CI: 0.88, 0.92) (Fig. 2a). For experiencing any AE ≥ grade 2, the ECI was 0.54 (0.50, 0.58) at day 5 and 0.69 (0.65, 0.73) at day 10 (Fig. 2b). For any AE ≥ grade 3, the ECI was 0.20 (0.17, 0.23) at day 5, 0.28 (0.24, 0.32) at day 10, and 0.32 (0.27, 0.37) at day 15 (Fig. 2c).

MedDRA classification and HGAE that occur in greater than 10% of visits are presented in Table 3. The results indicate visits during which the subject experienced a given AE classification at least one time; subjects could have experienced more than one AE class during a single visit. During the majority of visits, subjects experienced AEs that were classified as fatigue (*n* = 370, 48.2%), nausea (*n* = 247, 32.2%), insomnia (*n* = 257, 33.5%), headache (*n* = 231, 30.1%), hypertension (*n* = 224, 29.2%), presyncope (*n* = 217, 28.3%), dyspepsia (*n* = 199, 25.9%), and back pain (*n* = 197, 25.7%). Presyncope (*n* = 212) and hypertension (*n* = 77) occurred in the majority of visits in which grade 2 and grade 3 was the HGAE experienced, respectively. Of the 77 visits with a grade 3 hypertension AE, 75 had hypertension as a chief complaint upon arrival.

**Table 3:** Number of visits with AEs. Visits may have more than one AE type

		Highest Grade AE				
MedDRA term	Total n (%)	1	2	3	4	5
Fatigue	370 (48.2)	165	191	14	0	0
Insomnia	257 (33.5)	171	49	37	0	0
Nausea	247 (32.2)	207	40	0	0	0
Headache	231 (30.1)	157	44	30	0	0
Hypertension	224 (29.2)	56	91	77	0	0
Presyncope	217 (28.3)	0	217	0	0	0
Dyspepsia	199 (25.9)	190	9	0	0	0
Back pain	197 (25.7)	149	28	20	0	0
Pain in extremity	123 (16)	101	10	12	0	0
Abdominal pain	116 (15.1)	100	8	8	0	0
Diarrhea	109 (14.2)	90	11	8	0	0
Vomiting	96 (12.5)	76	15	5	0	0
Arthralgia	93 (12.1)	75	8	10	0	0
Palpitations	89 (11.6)	87	2	0	0	0

MedDRA classification and AE grade of the total number of AEs are presented in (Additional file 1: Table S1). There were a total of 5961 AEs. Of these, 75.3% were AE1, 19.6% were AE2, 5.0% were AE3, and 0.0003% were AE4. The most common events were the same as those that occurred in the majority of visits in Table 3.

### Serious adverse events

There were two (0.002% of visits) SAEs that required hospitalization. One was a grade 3 dehydration event that occurred on fasting day 3 in a 73-year-old, male patient. The patient developed headache, fever, and increased blood pressure. He was taken to an emergency facility, where he received antibiotics for potential upper respiratory infection and intravenous (IV) electrolytes for dehydration and was kept under inpatient observation for 3 days. The patient fully recovered and returned to TNHC. The other was a grade 4 hyponatremia event that occurred on fasting day 9 in a 70-year-old, male patient. The patient developed difficulty with speech and was immediately transported to an emergency facility by ambulance. Upon evaluation at the emergency facility, the patient was diagnosed with hyponatremia, administered IV electrolytes, and kept under inpatient observation for 4 days. The patient fully recovered but did not return to TNHC.

## Discussion

To our knowledge this is the first peer-reviewed assessment of AEs experienced during medically supervised, water-only fasting. We found that in the majority of visits (65.8%) the HGAE experienced was mild (grade 1) to moderate (grade 2) and there was no AE in 6.5% of visits. A severe but not life threatening AE (grade 3) was the highest grade in 27.6% of visits, and a life threatening AE (grade 4) was the highest grade in 1 visit. An SAE occurred in only 2 visits, and there were no deaths. HGAE and fast duration were positively correlated. Subjects also had less severe AEs during refeeding in comparison with fasting. The degree to which these observations are due to treatment (i.e., fasting) emergent AEs, pre-existing conditions, longer observation times, and/or other factors is unclear. Further research is necessary to better understand these relationships.

AEs that were commonly experienced during visits, including nausea, headache, insomnia, back pain, dyspepsia, and fatigue, were predominately mild, grade 1 events and are reactions that are known to occur during fasting [1, 2]. The exceptions were presyncope and hypertension. Presyncope is also known to occur in response to fasting, [1] and is always defined as a moderate, grade 2 event [38]. Hypertension was the largest category of grade 3 events – both for visits in which the highest event was grade 3 and for the total number of individual grade 3 events – and is not reported to occur in response to fasting. Conversely, water-only fasting has been shown to reduce blood pressure in hypertensive patients [5, 6]. Indeed, we found that in 97% of visits with a grade 3 hypertension AE, the patient had hypertension as a chief complaint. This suggests that the grade 3 hypertensive events occurred in patients being treated for hypertension and that water-only fasting is unlikely a causal factor in the participant’s high blood pressure.

A limitation in our assessment of water-only fasting safety is that patient notes were not recorded with the intent to assess AEs. This resulted in data incompleteness and the potential for other errors (e.g., inaccurate terminology usage and patient prompting). Data incompleteness prevented conclusive assignment of attribution, identification of treatment emergent AEs, and analysis of confounders. This could lead to an overestimation of AEs attributable to water-only fasting as seems to be observed for hypertensive events in patients with hypertension as their chief complaint. Additionally, this study reviewed charts from a single site and the findings are therefore specific to TNHC and may not be applicable to other fasting centers. However, to our knowledge, there are currently no other facilities conducting medically supervised, water-only fasting. Furthermore, the data were abstracted by a single clinician (J.S.F.) trained in MedDRA terminology and CTCAE grading but who was not blind to the study question. To address this limitation, we implemented multiple data consistency queries and performed an intra-rater assessment on a random sample of daily logs. Lastly, although we have quantified the safety profile of water-only fasting in the TNHC population, any judgment of the relative safety of water-only fasting is necessarily descriptive in nature as we did not identify a comparison group(s).

Our purpose in highlighting the Wilhelmi de Toledo et al. article [17] was not to criticize a seminal paper on fasting guidelines but rather to underscore two important points. The first is that even though fasting has been utilized by humans for millennia and the medical community for at least a century it was not until 2013 that peer-reviewed fasting guidelines were published in the English language. These guidelines as well as an increase in scientific literature on the benefits of various types of fasting support the credibility and emerging acceptance of this intervention [4, 53–55]. The second point is that within the fasting community – not to mention the greater medical community – there are claims, unsubstantiated by research, about the utilization and safety of water-only fasting (i.e., that it is not utilized and unsafe). Although controlled clinical trials are lacking, water-only fasting is utilized therapeutically and the data from this retrospective safety study suggests that adverse events experienced during medically supervised, water-only fasting are tolerable.

## Conclusions

Overall, our data indicate that the majority of AEs experienced during medically supervised, water-only fasting are mild to moderate in nature and are known reactions to fasting. This suggests that the TNHC protocol can be safely implemented in a medical setting with minimal risk of a severe or SAE. This study also provides a basis by which further research into the safety and efficacy of this intervention can be conducted.

## Additional file

Additional file 1: Supplementary Methods. (DOCX 63 kb)

## Abbreviations

AE: Adverse event
CI: Cumulative incidence
CTCAE: Common Terminology Criteria for Adverse Events
ECI: Estimated cumulative incidence
HGAE: Highest grade adverse event
HLT: Highest level term
IQR: Interquartile range
IV: Intravenous
LLT: Lowest level term
MedDRA: Medical Dictionary for Regulatory Activities
SAE: Serious adverse event
SOC: System organ class
TNHC: TrueNorth Health Center
VAS: Visual analog scale
