# Supplementary material for: Is fasting safe? A chart review of adverse events during medically supervised, water-only fasting
Source: BMC Complement Altern Med. 2018 Feb 20;18:67. doi: 10.1186/s12906-018-2136-6 (PMC5819235; doi:10.1186/s12906-018-2136-6)
Supplement: Supplementary file 1 — Supplementary Methods. (DOCX 63 kb) [file 12906_2018_2136_MOESM1_ESM.docx]

**ADDITIONAL FILES**

**Supplementary Methods**

**TrueNorth Health Center water-only fasting protocol**

**Pre-fast**

*Preparation (at least 2 days prior)*

- Eliminate all recreational drugs (i.e., coffee, alcohol, nicotine, etc.).
- Eliminate grains, legumes, dairy, meat, sugar, oils, salt and all processed foods.
- Consume only raw fruits and vegetables and steamed vegetables.

*Examinations*

- Detailed patient history
- Comprehensive physical exam, including height, weight, temperature, blood pressure, pulse
- Basic neurological and psychological status
- Complete blood count (CBC) with differential
- Comprehensive metabolic panel (CMP)
- Urine analysis (UA)
- Additional tests as clinically indicated

*Medications and Supplements*

- Taper off and discontinue all medications that can be safely discontinued.
- Replacement medications (i.e., thyroid medications) can be continued at reduced dosage.
- Taper off and discontinue all supplements.
- If medications cannot be discontinued modified fasting is indicated.

**Fast**

*Therapy*

- Steam-distilled water (min. 40 ounces/day)
- Limit excessive physical activity.
- Rest and relaxation
- Termination: condition resolves; patient requests; or medically necessary

*Examinations*

- Symptom inquiry (2X/day)
- Blood pressure and temperature and pulse (2X/day)
- CBC, CMP, UA (1X/week)
- Additional tests as clinically indicated (as needed)

*Treatments & Education Options*

- Medical
- Psychological
- Chiropractic
- Naturopathic
- Massage and body work
- Acupuncture
- Meditation
- Gentle yoga
- Daily lectures
- Cooking demonstrations
- Educational DVDs

**Post-fast**

*Standard Refeeding**

- Duration is half of the fast length.
- Chew food thoroughly.
- 1 day on each phase for every 7-10 days of water-only fasting

Phase 1: Fruit and vegetable juice (4X/day)

Phase 2: Raw fruits (except citrus) and raw juicy vegetables (3X/day)

Phase 3: Raw fruits and vegetables, steamed vegetables (3X/day)

Phase 4: Raw fruits and vegetables, steamed vegetables, grains, soups, and nuts (up to 1 oz/day) or avocado (half/day) (3X/day)

Phase 5: Unrestricted plant food diet free of added sugar, oil, and salt (3X/day)

* Sensitive refeeding plans optional

*Examinations*

- Twice daily check-ins with clinical staff
- Exit appointment with clinician

*Miscellaneous*

- Bowel movements begin gradually over course of refeeding; guidance provided.
- Increase physical activity slowly.

**Relative Contraindications**

- Cachexia
- Anorexia
- Severe liver or kidney disease
- Advanced cerebral vascular insufficiency
- Certain cancers
- Medium-chain acyl CoA dehydrogenase deficiency
- Certain psychological disorders
- Pregnancy or nursing
- Porphyria
- Higher-grade cardiac arrhythmias
- Active gastric ulcer disease

**Serious Complications**

- Severe electrolyte imbalance

**Minor Reactions**

- Mild hypoglycemia
- Electrolyte disturbances
- Headache
- Acute back pain
- Nausea
- Vomiting
- Muscle cramps
- Insomnia
- Orthostatic hypotension
- Stomach and reflux symptoms

**SUPPLEMENTARY TABLE**

| **Table S1. Total AEs grouped by System Organ Class.** | | | | | | | |
| --- | --- | --- | --- | --- | --- | --- | --- |
|  | |  | **AE Grade** | | | | |
| **MedDRA term** | **Total n (%)** |  | **1** | **2** | **3** | **4** | **5** |
| **Cardiac disorders** | | | | | | | |
| Palpitations | 94 (1.6) |  | 92 | 2 | - | - | - |
| Ventricular arrhythmia | 2 (0) |  | 2 | 0 | 0 | 0 | 0 |
| **Gastrointestinal disorders** | | | | | | | |
| Nausea | 328 (5.5) |  | 284 | 44 | 0 | - | - |
| Dyspepsia | 252 (4.2) |  | 242 | 10 | 0 | - | - |
| Abdominal pain | 158 (2.7) |  | 136 | 12 | 10 | - | - |
| Diarrhea | 146 (2.4) |  | 122 | 16 | 8 | 0 | 0 |
| Vomiting | 123 (2.1) |  | 101 | 17 | 5 | 0 | 0 |
| Flatulence | 85 (1.4) |  | 83 | 2 | - | - | - |
| Gastrointestinal pain | 68 (1.1) |  | 57 | 9 | 2 | - | - |
| Constipation | 54 (0.9) |  | 42 | 8 | 4 | 0 | 0 |
| Bloating | 47 (0.8) |  | 47 | 0 | 0 | - | - |
| **General disorders and administration site conditions** | | | | | | | |
| Fatigue | 550 (9.2) |  | 305 | 230 | 15 | - | - |
| Pain | 77 (1.3) |  | 61 | 13 | 3 | - | - |
| Malaise | 31 (0.5) |  | 24 | 7 | - | - | - |
| **Metabolism and nutrition disorders** | | | | | | | |
| Dehydration | 9 (0.2) |  | 7 | 1 | 1 | 0 | 0 |
| Hyponatremia | 5 (0.1) |  | 1 | - | 2 | 2 | 0 |
| **Musculoskeletal and connective tissue disorders** | | | | | | | |
| Back pain | 273 (4.6) |  | 218 | 35 | 20 | - | - |
| Pain in extremity | 161 (2.7) |  | 131 | 17 | 13 | - | - |
| Arthralgia | 131 (2.2) |  | 104 | 16 | 11 | - | - |
| Buttock pain | 75 (1.3) |  | 67 | 5 | 3 | - | - |
| Neck pain | 69 (1.2) |  | 59 | 7 | 3 | - | - |
| Chest wall pain | 50 (0.8) |  | 46 | 2 | 2 | - | - |
| **Nervous system disorders** | | | | | | | |
| Headache | 327 (5.5) |  | 235 | 60 | 32 | - | - |
| Presyncope | 253 (4.2) |  | - | 253 | - | - | - |
| Dizziness | 51 (0.9) |  | 44 | 4 | 3 | 0 | 0 |
| Syncope | 12 (0.2) |  | - | - | 12 | - | - |
| **Other** | | | | | | | |
| Other | 1356 (22.7) |  | 1235 | 104 | 17 | 0 | 0 |
| **Psychiatric disorders** | | | | | | | |
| Insomnia | 358 (6) |  | 252 | 66 | 40 | - | - |
| **Renal and urinary disorders** | | | | | | | |
| Hematuria | 43 (0.7) |  | 41 | 2 | 0 | 0 | 0 |
| Renal calculi | 1 (0) |  | 0 | 1 | 0 | 0 | 0 |
| **Respiratory, thoracic and mediastinal disorders** | | | | | | | |
| Sore throat | 75 (1.3) |  | 74 | 1 | 0 | - | - |
| Postnasal drip | 48 (0.8) |  | 45 | 3 | - | - | - |
| **Skin and subcutaneous tissue disorders** | | | | | | | |
| Rash acneiform | 78 (1.3) |  | 73 | 5 | 0 | 0 | 0 |
| Rash maculo-papular | 10 (0.2) |  | 8 | 2 | 0 | - | - |
| **Vascular disorders** | | | | | | | |
| Hypertension | 561 (9.4) |  | 252 | 214 | 95 | 0 | 0 |
| **Total** | | | | | | | |
| Total | 5961 |  | 4490 | 1168 | 301 | 2 | 0 |
| Includes MedDRA terms that were experienced in greater than 5% of the visits. A single dash (-) indicates CTCAE grade is not available. | | | | | | | |

**SUPPLEMENTARY FIGURE**


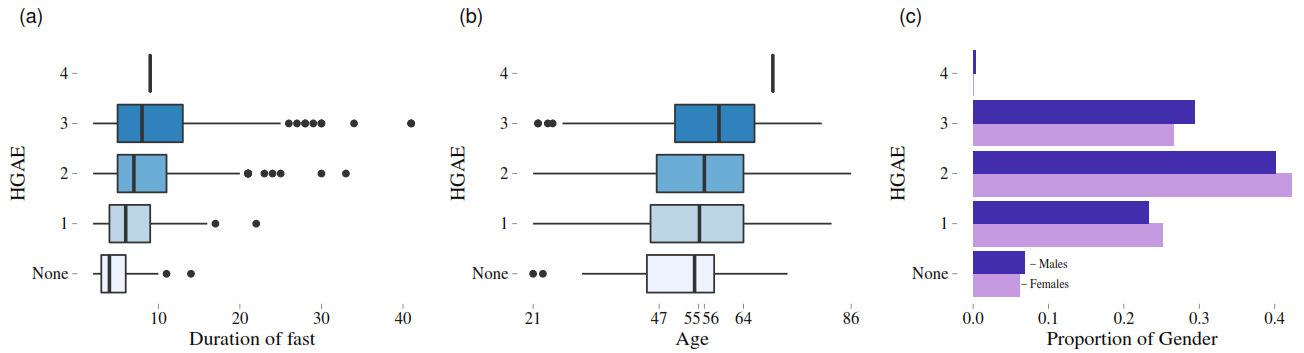


**Figure S1.** Highest grade adverse event (HGAE) during each visit by gender and age. Box plots (boxes show interquartile range and median, whiskers show 10^th^ and 90^th^ centiles, and circles are outliers) of HGAE and fast duration (a) and age (b). Spearman’s coefficient (ρ = 0.28) and paired t-test (p = <0.001) indicate that fast duration is positively correlated with HGAE. Spearman’s coefficient (ρ = 0.11) and paired t-test (p = 0.002) indicate that age is positively correlated with HGAE. Bar graph of the proportion of males and females with HGAE (c). There was no difference between genders for HGAE (p = 0.628 by Fisher’s exact test).
